# Supporting early-career mentorship for women in Health Policy and Systems Research: a vital input to building the field

**DOI:** 10.1093/heapol/czaa105

**Published:** 2020-11-09

**Authors:** Aku Kwamie, Nanuka Jalaghonia

**Affiliations:** c1 Alliance for Health Policy and Systems Research, World Health Organisation, 20 Avenue Appia, 1211 Geneva 27, Switzerland; c2 Health Systems Global, Curatio International Foundation, 0179 Kavsadze street. 3, Office 5, Tbilisi, Georgia

## Why support for early-career women researchers from low- and middle-income countries is still needed to publish their work

Peer-reviewed publications are an important part of the research domain. They communicate scientific work, foster recognition across academic communities and are important for career progression. ‘Publish or perish’ is a universally accepted adage which speaks to the pressure that researchers face to produce findings that are considered relevant and rigorous by the audience of professional colleagues. For early-career researchers in particular, this pressure is even more acute, as the act of being published represents the initiation of a foundation of good scholarly standing (O'Brien *et al.*, 2019).

In the field of health policy and systems research (HPSR), the growing body of peer-reviewed literature on theoretical frameworks, research methods and empirical data in varied topical areas marks the steady evolution and maturation of the field in recent years. Evidence shows that from 1990 to 2015, there has been a five-fold increase in HPSR publications annually (Alliance for Health Policy and Systems Research, 2017). In the same period, publications from low- and middle-income country (LMIC)-based lead authors have also increased (El-Jardali *et al.*, 2011; Defor *et al.*, 2017), demonstrating that a greater proportion of HPSR is being undertaken and produced by LMIC researchers. This is an important development.

However, globally, women are known to make up <30% of researchers, and while this proportion is increasing, women continue to publish less than their male counterparts (Shannon *et al.*, 2019). The gender gap in publishing is even more pronounced in LMICs (Morgan *et al.*, 2019). In the past 40 years, the gender gap in research publication has persisted, with men not only producing more than women but also being over-represented among the top ‘producers’ (Nygaard and Bahgat, 2018). This has significant consequences in academia, where career promotion systems are often based on the prestige of publishing, over teaching and other services (Aiston and Jung, 2015), and also influences policy and practice, where ideas and concepts are implemented. Evidence further shows that gender differences exist in the way male- and female-authored papers are self-presented, with male-authored papers being more ‘promotional’, a difference that is even more pronounced in higher-impact journals (Lerchenmueller *et al.*, 2019). While exact figures of women publishing in HPSR are not known, there is no reason to believe that they vary much from the broader scientific field, and calls have been made to proactively retain and promote women in global public health, especially from LMICs (Downs *et al.*, 2016). There is very much a need to systematically encourage opportunities for LMIC early-career researchers who are women to publish their work. There is equally the need to provide mentorship and guidance as first-time authors navigate their way through the process of publication.

## Intensifying efforts to support early-career women to publish: the Alliance, Health Systems Global and HPP

Over its 20-year existence, the Alliance for Health Policy and Systems Research (the Alliance) has always held knowledge generation and strengthening capacities to disseminate and use HPSR as core strategic objectives to achieve its mandate of strengthening LMIC health systems through HPSR. Health Systems Global (HSG), the member-driven global society for HPSR established in 2012, brings together policymakers, practitioners, researchers and civil society to facilitate (with their collective capacity) a diverse and unified community, and promote learning and knowledge translation, in order to strengthen health systems.

Both the Alliance and HSG demonstrate their commitment to the advancement and support of individual and institutional capacities for the conduct and uptake of HPSR in their current strategic plans. As constituencies, women and early-career researchers have been imperative to both the Alliance and HSG. Since 2014, the Alliance has instituted a gender equity policy, where research teams applying for grants should consist equally of women and men. This has resulted in near parity in publication of Alliance-supported authors (see the Alliance publications database), and the Alliance continues its investments to redress gender disequilibria in HPSR. Since its establishment, HSG has operationalized gender parity in its governance structures, in its Thematic Working Groups (TWGs) and among participants of the Global Symposia on Health Systems Research (see the Symposium Evaluation Report, Health Systems Global (2018)). Furthermore, it recently developed its capacity-strengthening strategy to address the needs expressed by its membership. Online surveys conducted in 2017 and 2019 identified areas of focus as: the capacities of young and LMIC-based researchers; mentorship and peer-to-peer interaction; and support for publication. HSG has previously partnered with science journals to deliver webinars to its membership, aimed at improving their understanding of the publication process (see the BMC-HSG webinar series).

In 2018, the Alliance launched a mentorship programme to connect early-career women in LMICs working in HPSR with mentors who could guide them through the publication process, from finalizing manuscripts to submission and responding to reviewer comments. The objectives of the programme were to increase the numbers of early-career women from LMICs getting published, to strengthen their skills in producing strong manuscripts and to foster their development through the process. Following this initial cohort of 10 mentees in 2018, the Alliance and HSG, in partnership with *Health Policy and Planning*, came together to launch a call for a special issue of *Health Policy and Planning*, and to support a second cohort of early-career women authors. The call for applications posted on the HSG website attracted nearly 4000 unique visitors, making it HSG’s second most-visited page. Two hundred and fifty potential candidates applied, and 22 mentees were accepted into the programme to be matched with mentors to work towards the submission of their papers. This indicates a great appetite for such initiatives.

## Learning in order to provide better mentorship models

While this supplement presents the papers of first-time women authors from LMICs that were part of the 2019 cohort, there remains a great need not only to increase the number and scale of publication mentorship programmes like this one but additionally to study why and how they do (or do not) work, for whom and in which contexts. Impediments to publication for early-career women range from gendered roles in personal life to structural barriers that configure the professional sphere. Furthermore, while diverse approaches to strengthening HPSR capacities in LMICs at individual and institutional levels have been implemented, there are no definitive answers as to which strategies are the most effective. Longer-term efforts to enhance global health research mentorship have highlighted both mentor and mentee motivations, alignment of expectations, cultural competencies and institutional support as attributes which contribute to effective mentoring experiences that lead to mentee persistency, science identity and research self-efficacy (Lembani *et al.*, 2016; Pfund *et al.*, 2016; Charron *et al.*, 2019). Hamer and colleagues further describe the development of competencies such as maintaining effective communication, addressing diversity, fostering independence, promoting professional development and integrity, overcoming resource limitations and fostering institutional change as effective supports to mentorship. Special efforts to encourage younger generations of women researchers also include increasing visibility of female academics and priority schemes for women, and creating enabling institutional environments that convey flexibility, reflexivity and active seeking of female talent (Hamer *et al.*, 2019). Additionally, Pfund *et al.* (2016) focus on the mentor–mentee relationship itself, noting that mentoring takes place in a social context informed not only by mentor and mentee attributes but also by the specific discipline in which the mentoring takes place—an important consideration given the multi-disciplinary nature of HPSR. Yet, it remains unclear as to whether every attribute needs to be present in the mentoring relationship, or even which types of relationships (i.e. dyads, groups or networks) or modes (formal/informal, short term/long term, face to face/virtual) are the most effective (Pfund *et al.*, 2016).

We (the Alliance and HSG) conducted a preliminary survey of the mentors and mentees who participated in this programme. Though our findings are limited by the small sample size (*n* = 37), they do resonate with the above-mentioned factors for effective mentorship, and include: (1) professional development opportunities for TWG members to actively contribute to HSG through mentoring, and for first-time authors to publish in a prestigious journal; (2) matching mentors and mentees based on areas of interest; (3) incentives for both mentors and mentees (including registration for the next Global Symposium on Health Systems Research); (4) clarity of mentor–mentee roles and responsibilities through a signed agreement; and (5) conflict of interest and co-authorship guidance. We also note the limitations that the programme faced, in particular its short timelines and, in some cases, methodologically weak initial manuscripts and language barriers. Programmatic recommendations to improve and scale up these efforts include an initiating webinar to ensure all mentor–mentee dyads have a similar starting point, and drawing on additional editorial services. Reflecting on our experience, having dedicated institutional oversight was essential to manage the pools of mentors and mentees, establish good working practices and ensure the timeliness of deliverables. This is similar to experiences elsewhere (Vasylyeva *et al.*, 2019). What is important in considering the scale-up of such initiatives to LMIC contexts is to take the long-term perspective in creating a mentorship ‘identity’ which builds on local strengths and social dynamics, while taking into account resource and institutional constraints (Lescano *et al.*, 2019). Consequently, research networks (either South–South or North–South) may be useful in this regard.

Finally, it is equally important to understand the individual-level transformations that arise for both mentors and mentees as a result of engaging in mentorship. Our intention is to explore this further. Deeper understanding of how providing mentorship opportunities to inform and improve ongoing support for early-career women researchers, especially those from LMICs, is critical. It is important to learn from both mentees and mentors. This will involve developing fresh conceptual models and generating empirical evidence. In this manner, the Alliance and HSG continue to learn together how to better shape initiatives to strengthen capacities and generate knowledge for the progression of the field of HPSR.
